# BRAF V600E Mutation in Malignant Melanoma—A Romanian Research Experience

**DOI:** 10.3390/medicina60030351

**Published:** 2024-02-20

**Authors:** Elena-Roxana Avădănei, Irina-Draga Căruntu, Irina Nucă, Raluca Balan, Ludmila Lozneanu, Simona-Eliza Giusca, Cornelia Amalinei

**Affiliations:** 1 Department of Morpho-Functional Sciences I–Histology, Pathology, “Grigore T. Popa” University of Medicine and Pharmacy, 16 University Street, 700115 Iași, Romania; elena.avadanei@umfiasi.ro (E.-R.A.); raluca.balan@umfiasi.ro (R.B.); ludmila.lozneanu@umfiasi.ro (L.L.); simonaelizagiusca@gmail.com (S.-E.G.); cornelia.amalinei@umfiasi.ro (C.A.); 2 Praxis Medical Investigation Laboratory, 35 Moara de Vant Street, 700376 Iași, Romania; irina.resmerita@umfiasi.ro; 3 Romanian Medical Science Academy, 1 I.C. Bratianu Boulevard, 030171 Bucharest, Romania; 4 Department of Mother and Child Medicine-Genetics, “Grigore T. Popa” University of Medicine and Pharmacy, 16 University Street, 700115 Iași, Romania; 5 Department of Pathology, “Sf. Spiridon” Clinical Emergency County Hospital, 1 Independentei Street, 700111 Iași, Romania; 6 Department of Histopathology, Institute of Legal Medicine, 4 Buna Vestire Street, 700455 Iași, Romania

**Keywords:** malignant melanoma, BRAF V600E mutation, clinicopathological characteristics

## Abstract

*Background and Objectives*: The most common mutation in malignant melanoma (MM) is the single-point mutation of v-raf murine sarcoma viral oncogene homolog B1 (BRAF) oncogene. Our study aims to evaluate BRAF V600E mutation, highlighting its frequency differences in primary versus metastatic MM. *Materials and Methods*: The study group comprised 133 patients diagnosed with MM in several county hospitals of the north-eastern region of Romania who have been assigned for investigation into BRAF V600E mutation in the private medical system. The material consisted of archived formalin-fixed paraffin-embedded (FFPE) blocks. BRAF V600E mutation was identified using the fully automated Idylla^TM^ BRAF mutation test system. *Results*: Out of the total of 133 cases, 78 cases were primary tumors, while 55 cases were metastatic MMs. Genetic analysis revealed the presence of BRAF V600E mutation in 66 cases (49.62%) and the wild-type genotype in 67 cases (50.37%). We found a statistically significant difference of the mutation frequency according to age (*p* = 0.0072). The mutated genotype was found in 45 cases out of 78 primary MMs (57.69%) and in 21 cases out of 55 secondary MMs (38.18%), with a statistically significant difference in favor of primary tumors (*p* = 0.0413). The correlations between the histopathological types, Clark’s level, Breslow index, ulceration, and lymphovascular invasion, respectively, and the mutated genotype were not statistically significant. BRAF V600E mutation was identified in 15 out of 40 secondary tumors with lymph node location (37.5%) and in 6 out of 15 secondary tumors with another location (40%) without statistically significant differences between the mutation frequency and the location of the secondary tumors. *Conclusions*: Our results support MM high genetic heterogeneity, pointing out the relationship between BRAF V600E mutation and several clinicopathological characteristics, in primary and metastatic MMs, stressing the importance of BRAF testing implementation in Romania.

## 1. Introduction

Malignant melanoma (MM) is one of the most aggressive human cancers [1]. The modern lifestyle and global atmospheric changes have lead to an increased exposure of the skin to ultraviolet (UV) rays, inducing an increased MM incidence [2]. MM ranks among the top 10 most common malignancies in most European countries [2], showing a high metastatic potential [1] and representing a significant burden for healthcare providers. The diagnosis of cutaneous MM has been established in the last decades by a spectrum of histopathological characteristics [3].

Histopathological examination has been considered, until recently, the “gold standard” of diagnosis, being complemented by a relatively reduced set of immunohistochemical markers. Deep changes in diagnosis have lately occurred due to the implementation of MM dermatological screening programs, leading to an increased detection of premalignant lesions. Nowadays, new and highly sensitive molecular tests are applied in malignancy diagnosis [1]. The worldwide sequencing of thousands of tumors has defined the genomic atlas of melanoma, included in the Cancer Genome Atlas Network [4]. It was demonstrated that the most common mutation in cutaneous MM, identified in nearly half of these tumors, is the single-point mutation of v-raf murine sarcoma viral oncogene homolog B1 (BRAF) oncogene in the exon 15/codon 600 [5,6]. BRAF is a serine/threonine kinase belonging to the rapidly accelerated fibrosarcoma (RAF) family [5,6,7,8]. The substitution of valine by glutamic acid at codon 600 (V600E) leads to constitutive activation of the kinase enzymatic activity of BRAF protein and downstream mitogen-activated protein kinases (MAPK) pathway. However, the incidence of BRAF mutation is approximately 20% and 6% in acral and mucosal melanoma, respectively, being much lower than that registered in cutaneous melanoma [9,10]. The neuroblastoma RAS viral oncogene homolog (NRAS) oncogene mutation in exon 3/codon 61 has been identified with a significantly lower frequency (approximately 20%) in cutaneous MM. Moreover, the receptor tyrosine kinase (KIT) gene is also mutated with a similar frequency (less than 15%) in MM [5,6]. The mutations of NRAS, neurofibromin 1 (NF1), and KIT genes are also responsible for MAPK pathway hyperactivation in MM. These mutations can be identified by different methods, such as Sanger, next-generation sequencing (NGS), and polymerase chain reaction (PCR), while microfluidic PCR is considered as a new and quick technique.

Alongside with the development of new molecular diagnosis techniques, the therapeutic approach of melanoma has registered a significant progress. About 10 years ago, vemurafenib and dabrafenib were approved by the Food and Drug Administration for the treatment of advanced melanoma with BRAF mutation. As a consequence, these two BRAF-targeted agents achieved a considerable increased response rate, from 5% to 48%, with a single-agent administration (vemurafenib) [11], with complete regression of the tumor registered in some patients [12,13,14]. BRAF inhibitors are now used in MM therapy, demonstrating their effectiveness by improving eligible patients’ life quality and prolonging their survival. Despite all these achievements, it remains unclear if BRAF mutation is associated with any specific melanoma stage.

A meta-analysis of the literature on this topic found inconsistent data with only 11 articles from a total of 603 articles meeting the inclusion criteria [15]. This study concluded that BRAF mutations are highly unpredictable, can be retained or lost in secondary lesions, and may occur only in metastatic lesions [15]. Additionally, the polyclonal model of melanoma, in which subclones with different BRAF status may coexist in the same melanoma lesion, are supported by other studies [16,17,18]. In addition, when considering treatment with kinase inhibitors, they point out the need for biopsy and subsequent BRAF analysis of a metastatic lesion [19].

Recently, an extensive multidisciplinary meeting of experts from the European Dermatology Forum (EDF), the European Academy of Dermatology and of Dermato-Oncology (EADO), and the European Organization for the Research and Treatment of Cancer (EORTC) provided the agreed recommendations for the diagnosis and therapeutic management of MM patients [20]. Based on clinical experience and on literature review, a general consensus of requesting BRAF V600 mutation testing in patients with stage III MM has been agreed [20]. BRAF V600 mutation analysis is required for treatment decision guidance in patients with distant or inoperable regional metastases and in patients with resected high-risk stage III melanoma, given the approval of BRAF and MEK inhibitors in the adjuvant setting [20]. Supplementary to that, BRAF V600 mutation testing should be carried out in metastatic tissue in either distant or regional metastases. If metastatic tissue sampling is not feasible, analysis can be performed on primary tumor samples, as there is a high concordance rate of BRAF status between primary and metastatic melanoma lesions [15,19,20]. On the contrary, another study suggests that MM visceral metastases of the BRAF genotype are rather heterogeneous and cannot be predicted based on primary tumor findings [6].

Our study aims to evaluate BRAF mutation in different MM subtypes and to correlate the evidence of the mutation with clinicopathological parameters, highlighting BRAF mutation frequency differences in primary versus metastatic MM.

## 2. Materials and Methods

### 2.1. Patients and Tissue Samples

The study group comprised 133 patients diagnosed with MM in several county hospitals in the north-eastern region of Romania who had been assigned for investigation of BRAF mutation in the private medical system, in “Praxis Medical Investigation Laboratory”, Iasi, between January 2022 and October 2023. Out of the total of 133 cases, 78 cases were primary tumors, whereas 55 cases were de novo metastatic tumors. Informed consent for the biological material used for research, following the diagnosis, was obtained from all patients. The inclusion criteria for our study were the following: (i) patients diagnosed with primary and secondary melanomas, (ii) patients who had only surgical therapy, without any adjuvant therapy, and (iii) patients referred for BRAF V600 mutation testing.

The material consisted of archived formalin-fixed paraffin-embedded (FFPE) blocks including tumor tissue collected for diagnosis. All cases were reevaluated using histological slides stained with hematoxylin and eosin (H&E) by assessing the morphological patterns and quantifying the percentage of tumor cells within the entire tumor volume. Tumor–normal cell ratio was determined by counting the nuclei in five 40x magnification microscopy fields by a pathologist (ERA) and the result was multiplied by 100 to obtain the percentage of tumor cells, according to the molecular biology technique protocol [21]. According to the percentage of tumor cells, sections of different thicknesses were sectioned from FFPE blocks as follows: (1) a section of 5 µm thickness, if the neoplastic cells represented more than 10% and corresponded to an area ≥ 50 mm^2^, (2) a section of 10 µm thickness, if the neoplastic cells represented more than 10% and corresponded to an area ≥ 25 mm^2^, (3) multiple sections of 10 µm thickness, if the neoplastic cells represented more than 10% but corresponded to an area < 50 mm^2^, and (4) macrodissection, if the neoplastic cells represented < 10% of the tumor volume [21].

### 2.2. Genetic Test—Idylla™ BRAF Mutation Test

Tumor sections from FFPE blocks were analyzed by real-time PCR using the fully automated Idylla^TM^ BRAF mutation test system (Biocartis, Mechelen, Belgium) in order to identify BRAF mutations. The Idylla molecular diagnostics platform from Biocartis was chosen for analysis as it is an innovative and fully automated real-time PCR system. It employs disposable cartridges containing five separate PCR chambers, allowing the concurrent detection and measurement of up to 30 molecular markers. This method is an in vitro test based on extracted DNA used for gene amplification, followed by subsequent real-time PCR detection of the target gene of interest. The test comprises three allele-specific PCR reactions that enable identification of *BRAF* wild type (WT), *BRAF*-V600E/E2/D, or *BRAF*-V600K/R/M sequences. The detection limit was set at a minimum 25 mm^2^ FFPE tissue present in a 5–10 µm slide and a neoplastic cell amount of more than 50%.

We used a cartridge system that was able to detect BRAF variants in our study group. The genetic testing was performed according to the manufacturer’s recommendations. The tumor area that ranged from 25 mm^2^ to 50 mm^2^ was transferred into the cartridge. After the deparaffinization, DNA was automatically extracted before the amplification in the real-time system with specific fluorescent probes for BRAF mutations. Sufficient DNA input and quality, as well as a successful PCR amplification, were ensured by amplifying a reference sequence and placing a positive control in each PCR chamber, respectively.

Only a limited number of tests (3 from the total of 133 analyzed cases) had inconclusive results, probably due to excess DNA, paraffin, and melanin, as explained in the protocol by the manufacturers. The tests were repeated for these patients.

### 2.3. Statistical Analysis

Statistical data processing was performed using SPSS version 20 (IBM, Armonk, NY, USA) and Microsoft Excel 2016 (Microsoft, Redmond, WA, USA). Continuous variables were expressed as the mean and standard deviations and the categorical variables as numerical values and percentages. We checked the normal distribution of continuous values. Regarding age, the Gauss curve showed an obviously abnormal distribution in the study group; therefore, we applied non-parametric tests for the patients’ ages. The level of mutation and the clinicopathological characteristics were achieved using non-parametric specific tests (chi-square test). The correlation was considered significant when *p* < 0.05.

## 3. Results

### 3.1. Clinicopathological Characteristics

The study group showed an approximately equal distribution between genders, with 61 men (45.86%) and 72 women (54.13%). The patients’ ages ranged between 19 and 86 years, with an average of 59 ± 14.39 years, showing a relatively proportional distribution, with 64 patients (48.12%) being younger and 69 patients (51.87%) being older than the average group age.

#### 3.1.1. Primary Malignant Melanoma

A total of 71 cases (91.02%) out of 78 primary MMs had a cutaneous MM and the remaining 7 cases (8.98%) had MMs arising from the mucous membranes.

These tumors had various locations, as follows: almost half of the cases (37 cases—47.43%) were located on the trunk, 10 cases (10.82%) on the lower extremities, 9 cases (11.53%) on the plants, 9 cases (11.53%) on the upper extremities, 7 cases (8.97%) on the head, 2 of them affecting the eyeball (1 uveal and the other of the internal eye angle) and a tumor involving the uvula, while the other 6 cases were situated in unusual locations, such as the anal canal (a case—1.28%), genital region (labia) (2 cases—2.56%), or respiratory mucosa (3 cases—3.84%).

**Figure 1:**
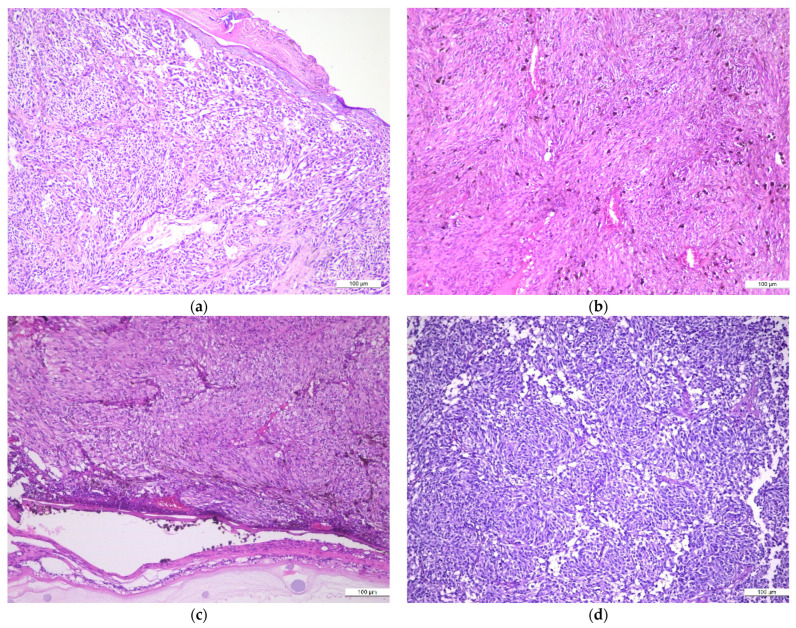
MM different histopathological patterns: (**a**) achromic epithelioid cell melanoma; (**b**) pigmented, spindle cell melanoma; (**c**) achromic spindle cell melanoma; (**d**) achromic spindle and clear cell melanoma; H&E, ×10.

Considering the histopathological subtypes, the primary cutaneous MMs were classified as NM in 51 cases (65.38%), LMM in 2 cases (2.56%), SSM in 8 cases (10.25%), and ALM in 9 cases (11.53%). Furthermore, we grouped the primary MMs into different subtypes, according to their morphological features, as follows: epithelioid type (37 cases—47.43%), with mixed epithelioid and spindle morphology (17 cases—21.79%), spindle/fusiform type (6 cases—7.69%), and with a polymorphous morphological picture (18 cases—23.07%). Figure 1 illustrates specific morphological patterns of the investigated MMs.

Based on the Clark system, primary MMs were classified into four categories: 2 cases (2.56%) as Clark’s II level, 15 cases (19.23%) as Clark’s III level, 31 cases (39.74%) as Clark’s IV level, and 17 cases (21.79%) as Clark’s V level, without any case diagnosed as Clark’s I level. The depth of invasion was evaluated using the Breslow quantification system. Based on this morphological system, the tumors were classified into four categories: <1 mm, 4 cases (5.12%), between 1.00 and 2.00 mm, 6 cases (7.69%), between 2.00 and 4.00 mm, 16 cases (20.51%), and >4.00 mm, 38 cases (48.71%). Overall, 31 (39.74%) out of the total 78 cases were ulcerated, this feature representing a poor prognostic factor. The lymphovascular invasion was identified in 17 cases (21.79%), 3 (3.84%) of them presenting additional perineural invasion; these parameters also representing unfavorable prognostic factors.

#### 3.1.2. Metastatic Malignant Melanoma

The majority of de novo metastatic MMs were located in the lymph nodes, 40 cases (72.72%) being identified in the sentinel lymph node and the remaining 15 cases (27.27%) having variable metastatic implants locations, as follows: 3 cases (5.45%) in the liver, 4 cases (7.27%) in the small intestine, 3 cases in the lung (5.45%), with 2 cases identified in the pleura and a case identified in the lung parenchyma, and a case (1.81%) in the parotid gland, 4 cases (7.27%) being secondary cutaneous tumors.

The main clinicopathological characteristics along with BRAF genotypes in the study group are summarized in Table 1.

### 3.2. Correlation between BRAF Mutated Genotype and Clinicopathological Characteristics

Genetic analysis of the study group revealed the presence of BRAF V600E/E2/D mutation in 66 cases (49.62%) and the wild genotype in 67 cases (50.37%). BRAF V600K/R/M mutation was not detected in any case of the study group.

The distribution of BRAF V600E mutation according to gender showed a relatively similar distribution, with 35 mutated cases in 72 male patients (48.61%) and 31 mutated cases in 61 female patients (50.81%), respectively. The statistical analysis did not show a statistically significant difference in relation to the patients’ gender (*p* = 0.9364) (Table 1).

The analysis of patients’ age distribution in the study group by comparison with the average age, which was 59 years, showed BRAF V600E mutation in 40 out of 64 patients (62.50%) with a lower age than the average and in 26 out of 69 patients (37.68%) with an older age than the group average. We registered a statistically significant difference in the mutation frequency according to age, clearly in favor of patients with younger ages than the group average age (*p* = 0.0072) (Table 1).

The mutated genotype was found in 45 cases out of 78 primary MMs (57.69%) and in 21 cases out of 55 secondary MMs (38.18%), with a statistically significant difference between the frequency of the mutated genotype, clearly in favor of primary tumors (*p* = 0.0413).

In relation to the histopathological subtype of the primary tumors, BRAF mutation was identified in 33 cases out of 51 NMs (64.70%), a case out of 2 LMMs (50%), 6 cases out of 8 SSMs (75%), and 3 cases out of 9 ALMs (33.33%). No statistically significant difference between the morphological subtype of the tumor and the presence of the mutated genotype was found (*p* = 0.2656) (Table 1).

Regarding the location of the primary tumor, 43 cases out of 71 primary cutaneous MMs (60.56%) and 2 cases out of 7 mucosal MMs (28.57%) exhibited a mutated genotype. The statistical analysis did not reveal any statistically significant difference between the tumor location and BRAF mutation (*p* = 0.2173) (Table 1).

According to Clark’s level, 2 cases labeled as Clark’s II level (100%) showed a wild genotype, whereas the mutated genotype characterized 9 out of 15 cases labeled as Clark’s III level (66.66%), 21 out of 31 cases with Clark’s IV level (67.74%), and 11 out of 17 cases with Clark’s V level (64.70%). Taking into consideration the Breslow index, the genetic analysis revealed the following distribution of the mutated genotype: 2 out of 4 cases (50%) with a depth of invasion < 1 mm, 4 out of 6 cases (66.66%) with a depth of invasion between 1 and 2 mm, 9 out of 16 cases (56.25%) with a depth of invasion between 2 and 4 mm, and 25 out of 38 cases (65.78%) with a depth of invasion > 4 mm. The statistical analysis showed no significant differences between Clark’s level or Breslow index and the mutated genotype (*p* = 0.3415 and *p* = 0.7107, respectively) (Table 1).

BRAF V600E gene mutation was confirmed in 18 out of 31 ulcerated primary MMs (58.07%) and in 27 out of 47 non-ulcerated primary MMs (57.44%), without statistically significant difference between ulceration and mutated genotype (*p* = 0.2269) (Table 1).

BRAF V600E mutation was identified in 15 out of 40 secondary MMs with lymph node location (37.5%) and in 6 out of 15 secondary tumors with another location (40%). The statistical analysis did not reveal any significant difference of the mutation frequency according to the location of the secondary tumors (*p* = 0.8874) (Table 1).

## 4. Discussion

Despite the significant progress in the development of treatment options, MM remains a leading cause of death. The understanding of the genetic, transcriptomic, and morphological spectrum of benign and malignant melanocytic neoplasia allowed the identification of new biomarkers with diagnostic, prognostic, and predictive values [22].

From this point of view, the identification of BRAF mutation in melanoma, in 2002, has opened up a new perspective in deciphering the carcinogenic mechanism and has provided the molecular basis for the development of new classes of therapeutic agents [5]. Over 50% of cutaneous melanomas harbor BRAF mutations, which can induce an enhancement of the kinase action that, consequently, leads to activation and augmentation of the downstream MAPK signaling pathway [7,12]. There is substantial evidence that alterations characteristic for melanocytic neoplasia development are regulated by the BRAF gene. This is supported by the identification of BRAF mutations in nevi and primary or metastatic melanoma, suggesting that this mutation is an early event in melanoma genesis [23], but the prognostic value of BRAF mutation requires further validation [24,25].

The lesions included in our study group showed the presence of BRAF mutations in 57.69% of cases diagnosed with primary MMs, in agreement with percentages reported in other studies [1,26,27]. On the other hand, the mutated BRAF genotype was identified in only 38.18% of cases of metastatic MMs. In this regard, the association with BRAF mutation still remains controversial in metastatic MMs [6], some studies reporting a concordant rate [27], with others showing significant discrepancies [28,29,30,31]. The statistical analysis identified a statistically significant difference in our study between the frequency of BRAF mutation in primary versus secondary MMs, in favor of the primary tumors. This finding suggests a loss of expression of the mutated alleles along with the metastatic progression of primary tumors. A plausible explanation for these controversial results could be the use of variable molecular techniques that exhibit different levels of sensitivity [32]. Another explanation is based on the heterogeneity of gene expression in melanoma that spreads far away from the primary site [18,33]. The novelty of our approach, aiming to evaluate BRAF mutation in primary and metastatic MMs, completes our preliminary communicated results, supporting the notion that BRAF mutation occurs most likely prior to the metastatic disease development [34].

Melanoma is a tumor that occurs in patients of any age. Accordingly, there was a wide range distribution, between 19 and 86 years, in our study group, with a relatively equal distribution of both genders, in agreement with literature reports [35]. BRAF mutation was more frequently identified in young patients, younger than our group average age, with statistically significant difference, when compared with BRAF mutation in older patients, consistent with literature data [36]. Several studies linked BRAF mutation with an unfavorable prognosis, its presence being associated with a reduced survival in stage IV melanoma, compared to patients showing the wild type [37,38,39]. However, this aggressive phenotype is also variable according to its intracellular location [40]. Furthermore, MMs harboring BRAF V600K mutation are considered as more aggressive than MMs with BRAF V600E mutation [41]. This characteristic suggests that the mutational spectrum of the BRAF gene should be fully described and that it may be considered as an essential criterion in MM diagnosis and therapeutic management [42]. No BRAF V600K mutation was detected in our study group, a finding consistent with a lower frequency of V600K mutations compared to V600E mutations reported in the literature [43].

Apart from the poor prognostic value, BRAF proto-oncogene mutation status is nowadays required to identify eligible patients for combined treatment with BRAF and MAPK inhibitors [35]. Therefore, our results can be directly translated into the therapeutic approach, young patients with a mutant genotype being eligible for therapy with anti-BRAF agents.

Literature reports support the prognostic value of the association between clinicopathological features and BRAF mutation [12,44]. However, the correlations between the histopathological types, Clark’s level, Breslow index, ulceration, and lymphovascular invasion, respectively, and the mutated genotype were not statistically significant in our study. Nonetheless, they have been validated as prognostic factors through the best practices guide implemented by experts in the field in an effort to adopt a unified approach to MM therapy [20]. Thus, our results do not support the correlation between the mutated genotype and the morphological profile.

The obtained data remain, at least for the moment, descriptive, the power of statistical analysis being limited by the relatively small number of cases comprising our study group.

A major clinical decision in MM therapeutic management is to perform sentinel lymph node biopsy [36]. There is compelling evidence that this procedure reclassifies the tumors from stage T1a, T1b, or TII directly to stage III disease, according to the American Joint Committee on Cancer [45,46]. This procedure, correlated with the identification of BRAF mutation, establishes the eligibility of patients for adjuvant systemic therapy [20,45]. This therapy may be represented by the association of dabrafenib and trametinib that improves the overall survival of patients at five years [47] or by pembrolizumab therapy that improves relapse-free survival in high-risk patients in stage III disease [48]. In an extensive follow-up examination involving adults who had not received prior treatment for metastatic melanoma and whose tumor tissue tested positive for V600 mutations, it was noted that BRAF inhibition with vemurafenib led to an improvement of survival rates [8,12,13,14]. Additionally, proactive management measures can effectively mitigate the impact of the adverse effects on patients, enabling continuous therapy despite its toxicity [49].

Sentinel lymph node biopsy detected 40 positive cases out of 55 de novo metastatic tumors (72.72%) in our study group. The genetic analysis of BRAF mutation, performed in order to identify among these patients which ones were eligible for anti-BRAF agents’ therapy, detected 15 patients (37.50%) with mutated variants out of 40 cases with tumoral lymph nodes, although in a rather small proportion of cases they were eligible for adjuvant therapy with BRAF inhibitors, already proven as an effective treatment by overall survival and relapse-free survival increase [20,48].

BRAF mutation study in MM has been a hot topic in dermatology and dermatopathology during the last two decades. Considering the current recommendations for the diagnosis and therapeutic management of MM patients [20], the major dilemma that arises is whether BRAF mutational testing maintains its value as a negative prognostic factor for primary MM. To the best of our knowledge, data on the association of BRAF mutation with MM are almost absent in Romania [50,51,52]. The major reason for this situation is the reduced access of patients to genetic investigations due to financial limitations, with repercussions in restricted options for targeted therapy. This is also reflected in the limitations of our study reporting the results obtained in the private health care system, which comprises a relatively small number of patients compared to other studies. However, our study was focused on the identification of BRAF mutation which completed the patient’s previous clinicopathological findings, monitored by the referring physician. We may consider that our study contributes to the broadening of the current framework of knowledge, with perspectives to optimize the diagnostic and therapeutic approach and, implicitly, to obtain a better understanding of the carcinogenic mechanism and MM specific behavior. Moreover, the main value of our study consists in providing information about the BRAF mutation profile in patients diagnosed with MM in Romania.

These considerations also support the necessity for an enhanced screening approach for skin melanoma in our nation employing molecular techniques. Although targeted therapy is a recent development in our region, BRAF mutations identification could guide the recommendation of these innovative medications.

## 5. Conclusions

The detection of BRAF mutation in MM using new sequencing techniques is mandatory in order to identify the eligibility of MM patients for the application of targeted therapies with BRAF inhibitors and to monitor their therapy responses or the development of therapy resistance. In this respect, the implementation of BRAF mutation analysis in the Romanian health system would improve MM patients’ prognosis.

Our results support the high MM genetic heterogeneity, pointing out the relationship between BRAF mutation and several clinicopathological characteristics in primary and metastatic MM.

## Figures and Tables

**Table 1:** Clinicopathological characteristics and BRAF V600E mutation status in MM.

Clinicopathological Characteristics	Total (#/%)	BRAF Genotype		*p* Value
		Mutated (#/%)	Wild Type (#/%)	
**Gender**				
Female	72/54.13	35/48.61	37/51.38	0.9364
Male	61/45.86	31/50.81	30/49.18	
**Age**				
≤59 years	64/48.12	40/62.50	24/37.50	0.0072 *
>59 years	69/51.87	26/37.68	43/62.31	
**Tumor type**				
Primary	78/58.64	45/57.69	33/42.30	0.0413 *
Metastatic	55/41.35	21/38.18	34/61.81	
**Histopathological types**				
NM	51/65.38	33/64.70	18/35.29	0.2656
LMM	2/2.56	1/50	1/50	
SSM	8/10.25	6/75	2/25	
ALM	9/11.53	3/33.33	6/66.66	
Not available	8/10.25	1/12.5	7/87.5	
**Metastases sites**				
Lymph nodes	40/72.72	15/37.5	25/62.5	0.8874
Other	15/27.27	6/40	9/60	
**Primary tumor location**				
Cutaneous	71/91.02	43/60.56	28/39.43	0.2173
Mucosal	7/8.97	2/28.57	5/71.42	
**Primary cutaneous melanoma** **Clark’s level**				
II	2/2.56	0/0	2/100	0.3415
III	15/19.23	9/60	6/40	
IV	31/39.74	21/67.74	10/3.22	
V	17/21.79	11/64.70	6/35.29	
Not available	13/16.66	4/30.76	9/69.23	
**Primary cutaneous melanoma** **Breslow thickness**				
<1.00 mm	4/5.12	2/50	2/50	0.7107
1.00–2.00 mm	6/7.69	4/66.66	2/33.33	
2.00–4.00 mm	16/20.51	9/56.25	7/43.75	
>4.00 mm	38/48.71	25/65.78	13/34.21	
Not available	14/17.94	5/35.71	9/64.28	
**Primary cutaneous** **melanoma ulceration**				
Present	31/39.74	18/58.06	13/41.93	0.2269
Absent	47/60.25	27/57.44	20/42.55	

#—number; %—percentage; * chi-square test (Pearson test)—significant when *p* < 0.05 (bold for significant value); ALM—acral lentiginous melanoma; LMM—lentigo maligna melanoma; NM—nodular melanoma; SSM—superficial spreading melanoma.

## Data Availability

The data used to support the findings of this research are available upon request to the authors.
